# Cross-sectional study on the dissociation of decision-making capacity for antipsychotic treatment and COVID-19 vaccination in individuals with schizophrenia

**DOI:** 10.3389/fpsyt.2023.1308666

**Published:** 2023-12-14

**Authors:** Stéphane Raffard, Sophie Bayard, Philippe Tattard, Yasmine Laraki, Delphine Capdevielle

**Affiliations:** ^1^Université Paul Valéry Montpellier 3, EPSYLON EA 4556, Montpellier, France; ^2^University Department of Adult Psychiatry, CHU Montpellier, Montpellier, France; ^3^FondaMental Academic Advanced Center of Expertise for Schizophrenia (FACE-SZ), Créteil, France; ^4^IGF, Université de Montpellier, CNRS, INSERM, Montpellier, France

**Keywords:** mental capacity, treatment, vaccination, antipsychotic, schizophrenia

## Abstract

**Objective:**

Decision-making capacity for treatment is impaired in schizophrenia but it remains unknown if schizophrenia affects distinct decision-making capacities differently.

**Methods:**

In this study, we assessed concomitantly two decision-making capacities (i.e., antipsychotic treatment and COVID-19 vaccination) in 27 schizophrenia patients. Sociodemographic variables, psychotic symptoms, global cognition and insight were also assessed.

**Results:**

We found that among individuals incompetent to consent to antipsychotic treatment, one-third had preserved capacity to consent to vaccination. No significant associations between the two different decision-making capacities were found. Psychotic symptoms and cognition were associated with the capacity to consent to antipsychotic treatment and to vaccination, respectively.

**Conclusion:**

Clinicians should be aware that capacity to consent to treatment is not unidimensional and vary across domains in individuals with schizophrenia. Being incompetent regarding one treatment does not mean to be incompetent for another treatment in this clinical population.

## Introduction

Clinicians have an ethical and professional obligation to obtain patient consent before initiating treatment. Capacity to consent is related to an individual’s ability to understand, appreciate, and manipulate information and to form rational decisions (1, 2). According to Appelbaum and Grisso (1), decision capacity is considered a four-dimensional concept, which includes (a) the understanding of the disclosed information, (b), the reasoning about the potential risks and benefits of their choices, (c) the ability to appreciate the nature of their situation and the consequences of their choices, and (d) the aptitude to express a choice (2). However, the assessment of competence to give informed consent to treatment is an important ethical and legal concern in some mental disorders such as schizophrenia (3). Indeed, schizophrenia is a mental disorder characterized by high prevalence of impaired decision-making abilities and accumulating evidence indicates that the capacity to make treatment decision is one of the most impaired decision-making capacities in schizophrenia compared to other mental disorders such as bipolar disorders (4). For example, several studies have shown that a large proportion of schizophrenia patients have diminished capacity to consent to antipsychotic treatments (5) or other medical treatment such as COVID 19 vaccines (6). However, it remains unknown whether the inability to consent for one specific treatment (e.g., antipsychotic) induces the same patient to be incompetent for another treatment (e.g., vaccine). This question raises an important clinical issue in this clinical population in which for many mental health professionals, a diagnosis of schizophrenia is equated with decisional incapacity (the status-based incompetence model) (7, 8), leading them to consider patients as generally incompetent regardless of the treatment (9).

The main aim of this study was to determine if competence to consent to two different treatments (antipsychotic vs. COVID-19 vaccines) are distinct or related in the same sample of individuals with schizophrenia. Our second aim was to explore the clinical determinants of each capacity to make treatment decisions.

## Materials and methods

### Participants

We assessed 27 outpatients with schizophrenia in the University Department of Adult Psychiatry of Montpellier, France between April 2021 and April 2022. The average chlorpromazine equivalent dose was 664 ± 417 mg/day. Among the patients, 90% were receiving a first-generation antipsychotic treatment, 8% a second-generation antipsychotic treatment, and 2% of patients were receiving a combination of such treatments. The proportion of patients with a score ≥ 25/30 on the MoCA was 37% (10). Inclusion criteria were: (a) age between 18 and 60 years, (b) a DSM-5 diagnosis of schizophrenia, and (c) adequate proficiency in French. Exclusion criteria for all participants were: (a) known neurological disease and (b) history of learning disability/developmental disorder.

### Ethical statement

This study was conducted in accordance to the ethical standards described by the Medical Research Involving Human Subjects Act (WMO) and was approved by the hospital’s institutional review board (IRB ID: 202100768). Written informed consent was obtained for all participants.

### Procedure

Sociodemographic and treatment information (for both antipsychotic medication and COVID 19 vaccination) were collected from the electronic medical records. Capacities to consent to antipsychotic treatment and COVID-19 vaccination were assessed during the same session by the same clinical psychiatrist. Psychotic symptoms were assessed with the Positive and Negative Syndrome Scale [PANSS; (11)], insight with the G12 item of the PANSS and cognition with the Montreal Cognitive Assessment [MOCA; (12)]. Due to the French vaccination strategy, patients were not offered vaccination at the psychiatric outpatient facility but in mass-vaccination centers, and retail pharmacies.

### Measures

Capacity to consent to antipsychotic treatment (CC-A) and COVID-19 vaccination (CC-V) were assessed with the MacArthur Competence Assessment Tool for Treatment [MacCAT-T; (13, 14) for the French validation]. We used two measures of capacity for each participant. A measure for COVID-19 vaccination (6) and a measure of competence to consent to antipsychotic medication (13). For each decision-making capacity: the patient’s understanding of the disorder/disease and treatment-related information (Understanding) was rated from 0 to 6; appreciation of the significance of that information for the patient, in particular, the benefits and risks of treatment (Appreciation), was rated from 0 to 4; the reasoning ability of the patient (Reasoning) was rated from 0 to 8; and ability of the patient to express a choice between the proposed treatment (i.e., antipsychotic or COVID-19 vaccine) and an alternative treatment (Expressing a choice) was rated from 0 to 2. Patients were divided into two groups based on ratings on the four subscales of the MacCAT-T and on the methodology of Elzakkers et al. (15). For all subscales a patient could rate poor (50% or less of the maximum rating on that subscale), intermediate (51–75% of the maximum rating) or good (76–100%). If a patient had a poor or intermediate rating on one or more of the four subscales, this patient was considered as having diminished mental capacity.

### Statistical analyses

Statistical analyses were performed with the Jamovi statistical computer software [The jamovi project (2021). jamovi. (Version 1.6) Retrieved from http://www.jamovi.org]. The χ2 and Mann–Whitney tests were, respectively, applied for qualitative and quantitative variables. Significance was set at a *p* value less than 0.05.

## Results

### Measures of CC-A and CC-V

Just under two-thirds of patients had a full vaccine status (58.8%). Note that around 80.1% of the general population had a full vaccination status (according to Sante Publique France https://www.santepubliquefrance.fr/dossiers/coronavirus-covid-19). This indicates that our sample of patients was under-vaccinated compared to the general population as shown in most of the studies on this topic and whatever the different phases of COVID-19 vaccination (16). As documented in Table 1, diminished mental capacity to consent to antipsychotic medication was observed and ranged from 66.7% for the Expressing a choice dimension to 77.8% for the Understanding dimension. With regard to the competence to consent to COVID-19 vaccination, these proportions varied from 18.5% (Expressing a choice) to 63% (Understanding).

**Table 1:** Sociodemographic and clinical characteristics of the sample (*n* = 27).

Patients characteristics	Value: mean (standard deviation) or %
Demographic variable	
Age	46 (12.8)
Gender, female	3
Years of education, mean	12.9 (3.18)
Full vaccine status	58.8%
Clinical variables	
Duration of the disease	26.4 (13.4)
PANSS^a^ total	72.8 (16.3)
PANSS positive	14.8 (5.2)
PANSS negative	18.8 (5.5)
PANSS general psychopathology	39.2 (7.6)
PANSS G12 item	3.7 (1.1)
Montreal Cognitive Assessment	22.1 (5.2)
MacCAT-T^b^, antipsychotic treatment	
Understanding	77.8%^c^
Appreciation	77.8%^c^
Reasoning	74.1%^c^
Expressing a choice	66.7%^c^
MacCAT-T^b^, COVID-19 Vaccination	
Understanding	63%^c^
Appreciation	59.3%^c^
Reasoning	55.6%^c^
Expressing a choice	18.5%^c^
Comorbidities	
Diabetes	18.5%
Pulmonary disease	29.6%
Cardiovascular disease	11.1%
One or more comorbidities	44.4%

a Positive and Negative Syndrome Scale.

b MacArthur Competence Assessment Tool for Treatment.

c Proportion of patients with diminished mental capacity.

**Figure 1:**
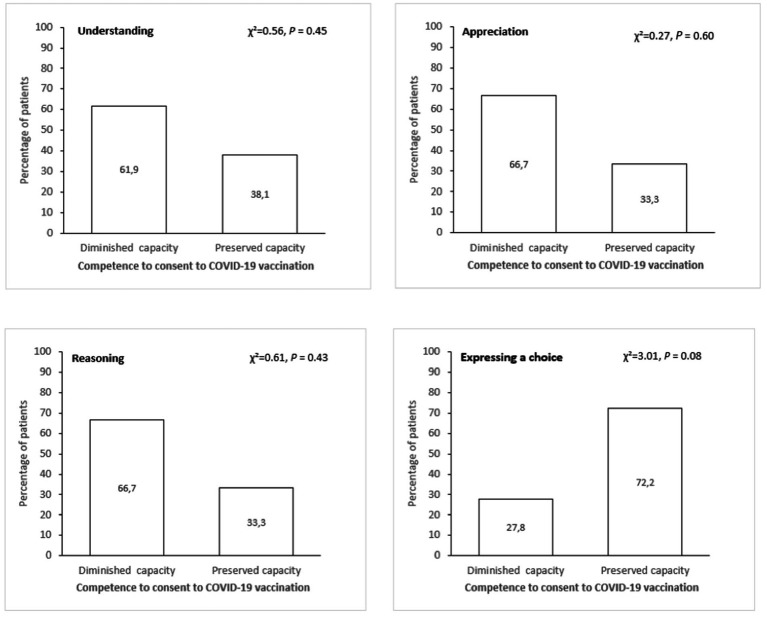
Percentage of patients with diminished or preserved capacity to consent to COVID-19 vaccination among patients with diminished capacity to consent to antipsychotic treatment according to Mac-CAT dimensions.

The percentage of patients with diminished or preserved capacity to consent to COVID-19 vaccination among patients with diminished capacity to consent to antipsychotic treatment according to MacCAT dimensions are documented in Figure 1. For the four dimensions of the MacCAT, we did not find a significant association between the capacity to consent to COVID-19 vaccination and the capacity to consent to antipsychotic medication (respectively, Understanding, *p* = 0.45; Appreciation, *p* = 0.60; Reasoning, *p* = 0.43; Expression a choice, *p* = 0.08). Descriptively, among patients who did not have the capacity consent to antipsychotic treatment, there is more than one-third with preserved capacity to consent to COVID-19 vaccine for Understanding (38.1%), Appreciation (33.3%) and Reasoning (33.3%) MacCAT dimensions.

### Associations with CC-A and CC-V

#### Clinical factors and psychotic symptoms

Regarding the clinical correlates of capacity to consent, we compared patients with poor capacity to consent to those with preserved capacity to COVID-19 vaccination and antipsychotic treatment on the following variables: disease duration, MoCA, PANSS total score and G12 PANSS item.

On the Understanding dimension patients with diminished CC-V had lower MoCA score compared to those with preserved capacity, respectively, *Mdn* = 19 versus 26 (U = 40, *p* = 0.012). No other differences were found for Appreciation, Reasoning and Expressing a choice MacCAT dimensions.

Regarding CC-A, patients with diminished CC-A had higher PANSS total scores compared to those with preserved capacity respectively, Understanding, *Mdn* = 78 versus 62.5 (U = 18.5, *p* = 0.04); Appreciation, *Mdn* = 78 versus 62.5 (U = 20.5, *p* = 0.05); Reasoning, *Mdn* = 80 versus 57, U = 6.5, *p* = 0.002; Expression a choice, *Mdn* = 81.5 versus 58 (U = 8.5, *p* = 0.003). A statistical tendency was noted on the item G12 insight for the latter dimension, *Mdn* = 4 versus 3, (U = 25.5, *p* = 0.07).

## Discussion

### Key findings

Our results indicated that in individuals with reduced capacity to consent to antipsychotic treatment (CC-A), approximatively one-third had preserved capacity to consent to COVID-19 vaccination (CC-V). This result is in accordance with those of Spencer et al. (17) who showed that people with schizophrenia commonly retain decision-making capacity for research despite lacking decision-making capacity for antipsychotic treatment. In addition, no relationships were found between the sub-dimensions of CC-A and CC-V indicating that decision-making capacity is not an unidimensional phenomenon in schizophrenia and vary across domains.

Secondly, we also found that whereas reduced CC-A was associated with higher level of psychotic symptoms and with poor insight, reduced CC-V was positively associated with cognition. These results are in line with the view that CC-A is mainly influenced by how patients with schizophrenia acknowledge the presence or severity of their disease and consequently the need of an antipsychotic treatment, whereas other decision-making capacities (including decision-making capacity for other treatment than antipsychotic or research) are more related to cognitive abilities (13, 15).

In our study, and following the methodology of Elzakkers et al. (14), 77.8% of patients had diminished capacity to consent to antipsychotic treatment. The fact that most of our patients in our study were not considered as having the capacity to consent to antipsychotic treatment substantially differs from other studies (18). In most of the existing studies study using the MacCAT-T, and in order to create two groups (full and diminished mental capacity) most of the authors used a rating of 50% or less on a subscale to indicate a poor outcome and any rating over 50% as a good outcome (18). In the present study, for every subscale, a patient could rate poor (50% or less of the maximum rating on that subscale), intermediate (51–75% of the maximum rating) and good (76–100%). If a patient had a poor or intermediate rating on one or more of the four subscales, he was considered as having diminished mental capacity on the MacCAT-T. In other words, the more severe cut-off used in our study to consider a patient as having a diminished capacity to consent to antipsychotic treatment might explain this result.”

## Strengths and limitations

This study is the first to the best of our knowledge to compare two different decision-making capacities for two distinct medical treatments (i.e., antipsychotic medication and COVID-19 vaccination) in a sample of people with schizophrenia.

However, this study has limitations. First, the sample size is small. In addition, insight was measured using the G12 insight item of the PANSS, which does not allow to capture the multidimensional aspect of insight.

## Conclusion

For schizophrenia, clinicians should determine a patient’s competence in a task-specific manner, and avoid the generalisation that an impaired ability to consent to one specific treatment means that the patient is incompetent to properly consent to another treatment. In other words, although an individual with a diagnosis of schizophrenia has a reduced capacity to appropriately consent to an antipsychotic treatment, a careful reassessment for all other treatments offered may be important for the proper care of the patient. While great strides have been made to respect patients’ autonomy in the case of physical illnesses, this is not yet the case in the case of mental disorders, despite the fact that the MacCAT–T can be used to produce highly reliable judgments of capacity (19).

## Data availability statement

The raw data supporting the conclusions of this article will be made available by the authors, without undue reservation.

## Ethics statement

The studies involving humans were approved by CHU Montpellier institutional review board (IRB ID: 202100768). The studies were conducted in accordance with the local legislation and institutional requirements. Written informed consent for participation in this study was provided by the participants’ legal guardians/next of kin. Written informed consent was obtained from the individual(s) for the publication of any potentially identifiable images or data included in this article.

## Author contributions

SR: Conceptualization, Funding acquisition, Methodology, Project administration, Writing – original draft, Writing – review & editing. SB: Formal analysis, Writing – review & editing. PT: Investigation, Writing – review & editing. YL: Visualization, Writing – review & editing. DC: Conceptualization, Investigation, Project administration, Writing – review & editing.
